# "GINEXMAL RCT: Induction of labour versus expectant management in gestational diabetes pregnancies"

**DOI:** 10.1186/1471-2393-11-31

**Published:** 2011-04-20

**Authors:** Gianpaolo Maso, Salvatore Alberico, Uri Wiesenfeld, Luca Ronfani, Anna Erenbourg, Eran Hadar, Yariv Yogev, Moshe Hod

**Affiliations:** 1 Department of Obstetrics and Gynaecology, Institute for Maternal and Child Health - IRCCS Burlo Garofolo, Via dell'Istria 65/1 34137, Trieste, Italy; 2 Division of Maternal Fetal Medicine - Helen Schneider's Hospital for Women - Rabin Medical Center, Petah Tikva, Israel

## Abstract

**Background:**

Gestational Diabetes (GDM) is one of the most common complications of pregnancies affecting around 7% of women. This clinical condition is associated with an increased risk of developing fetal macrosomia and is related to a higher incidence of caesarean section in comparison to the general population. Strong evidence indicating the best management between induction of labour at term and expectant monitoring are missing.

**Methods/Design:**

Pregnant women with singleton pregnancy in vertex presentation previously diagnosed with gestational diabetes will be asked to participate in a multicenter open-label randomized controlled trial between 38+0 and 39+0 gestational weeks. Women will be recruited in the third trimester in the Outpatient clinic or in the Day Assessment Unit according to local protocols. Women who opt to take part will be randomized according to induction of labour or expectant management for spontaneous delivery. Patients allocated to the induction group will be admitted to the obstetric ward and offered induction of labour via use of prostaglandins, Foley catheter or oxytocin (depending on clinical conditions). Women assigned to the expectant arm will be sent to their domicile where they will be followed up until delivery, through maternal and fetal wellbeing monitoring twice weekly. The primary study outcome is the Caesarean section (C-section) rate, whilst secondary measurement4s are maternal and neonatal outcomes. A total sample of 1760 women (880 each arm) will be recruited to identify a relative difference between the two arms equal to 20% in favour of induction, with concerns to C-section rate. Data will be collected until mothers and newborns discharge from the hospital. Analysis of the outcome measures will be carried out by intention to treat.

**Discussion:**

The present trial will provide evidence as to whether or not, in women affected by gestational diabetes, induction of labour between 38+0 and 39+0 weeks is an effective management to ameliorate maternal and neonatal outcomes. The primary objective is to determine whether caesarean section rate could be reduced among women undergoing induction of labour, in comparison to patients allocated to expectant monitoring. The secondary objective consists of the assessment and comparison of maternal and neonatal outcomes in the two study arms.

**Trial Registration:**

The study protocol has been registered in the ClinicalTrials.gov Protocol Registration System, identification number NCT01058772.

## Background

GDM is one of the most common complications of pregnancy and its incidence is estimated at 7% [1]. Among women affected by GDM, an increased rate of C-section has been observed, mostly unjustified [2,3]. Recent studies identified a C-section rate among women with this condition as high as 35% [4], and indicated a probability of undertaking a C-section in this group around 1.5 times the probability among non-GDM women [5].

Furthermore, babies born from women with GDM are significantly more exposed to perinatal risk, mostly related to fetal macrosomia [6]. This clinical condition is in fact associated to an increased risk of intrapartum traumatic lesions and asphyxia [7]. The incidence of macrosomia or birth weight above the 90^th ^percentile is more common among these women, in comparison to the general population [8]. These newborns, because of their peculiar intrauterine growth pattern, are characterised by a trunk mass larger than the head and consequently are more exposed to shoulder dystocia, bone fractures and brachial plexus injury, possibly with permanent outcomes [9,10].

The American College of Obstetricians and Gynecologists recommends a caesarean section for women with GDM and estimated fetal weight > 4500 g [11]. The American Diabetes Association recommends that GDM by itself is neither an indication for C-section nor for induction of labour before 38 completed gestational weeks [12]. Pregnancy prolongation beyond 38 weeks increases the risk to develop fetal macrosomia, without reducing C-section rate. As a result, delivery within the 38^th ^week of pregnancy is advisable if there are not other specific obstetric recommendations to determine an alternative management [12].

The guidelines currently in use are mainly based on retrospective studies and the experiences of single hospitals in the context of maternal and perinatal outcomes. As a consequence, there is ample possibility of variation in the clinical management of these women (expectant, induction or elective C-section) if determined by health professionals' individual preference [1]. To the best of our knowledge, only a randomized clinical trial [13] has been carried out and it has been included in the most recent Cochrane review investigating this issue [6]. Results of this trial suggested that expectant management after 38 weeks of pregnancy reduced Caesarean delivery rate and also increased the prevalence of large for gestational age newborns and shoulder dystocia [13]. Despite the importance of these results, this study was flawed by several methodological limitations. The randomization method and allocation concealment were not reported [10] and the sample population included both women with insulin-requiring gestational diabetes and class B pre-gestational. In addition, few non-randomized clinical trials [14,15] and small number of observational studies [8,16-19] have been published on this issue, but obvious methodological limitations interfered from drawing any definitive conclusions.

Strong evidence, based on adequately designed prospective studies and randomized controlled trials, in favour or against the effectiveness and safeness of induction in women with GDM are missing [1,10]. In light of this clinical dilemma, we propose a multicenter randomized controlled trial comparing induction of labour at 38-39 gestational weeks to careful expectant monitoring.

## Methods/Design

The proposed research regards a multicentre open label randomized controlled trial in women with GDM between 38+0 and 39+0 gestational weeks. The study will be carried out by the GINEXMAL Study Cooperative Research Group including 9 Teaching Hospitals, 5 in Italy and 4 abroad. The Institute for Maternal and Child Health - IRCCS Burlo Garofolo (Italy) together with The Helen Schneider's Hospital for Women - Rabin Medical Centre (Israel) will be the coordinating centre of the research project.

A total sample of 1760 patients (N = 880 per intervention group) will be recruited. The sample size has been estimated to be sufficiently large to achieve significant results with concerns to the main outcome of the study. In particular, it will be able to demonstrate a difference between the two arms ≥ 6% (31% of C-section in the expectant group and 25% in the induction group; relative difference between the 2 groups equal to 20% in favour of induction [13]), considering α error equal to 5% and 80% power.

Randomization will be centralized and coordinated by the Epidemiology and Biostatistics Unit (IRCCS Burlo Garofolo, Italy) using a computer-based method. The randomization list will be blocked (using blocks with a size of 10) and stratified by centre. The allocation concealment will be guaranteed through the use of closed opaque envelopes, consecutively numbered. In each envelope a patient's allocation group will be indicated, based on randomization. The study will be an open label trial because of the practical impossibility to blind either health professionals or patients to the allocation group.

Medical personnel directly involved in the management of the patient, will open the first available envelope and assign the patient to the corresponding randomization group (experimental group: induction of labour or control group: expectant management). The recruitment phase of the study will last 2 years.

Women older than 18 years of age with a singleton pregnancy in vertex presentation diagnosed with GDM in their current pregnancy will be eligible. Gestational diabetes mellitus is defined as carbohydrate intolerance, with onset or first recognition during pregnancy [20,21]. Diagnosis will be based upon abnormal 50 gr Glucose Challenge Test (GCT) (> 140 mg/dl) followed by 2 abnormal indices in the Oral Glucose Tolerance Test (OGTT) (according to Carpenter & Coustan Criteria). Women with GCT > 200 mg/dl will undergo 100 gr OGTT as well. If prior to the beginning of the study initial HAPO study consensus is available [21], diagnosis of GDM will be based on these criteria. Patients characterised by the mentioned eligibility criteria (Table 1) will be asked to take part in the study during a consultation at the Outpatient clinic at approximately 35 weeks of gestational age. At the consultation the patient will first have the protocol explained verbally. In order to give the individual an opportunity to consider the proposed proceedings, they will be left with a paper copy of the protocol for a short period of time. If the patient expresses a desire to take part, she will then be referred to the Day Assessment Unit between 38+0 and 39+0 weeks of gestational age.

**Table 1:** Inclusion and Exclusion Criteria

Inclusion Criteria
Maternal age ≥ 18 years
Singleton pregnancy in vertex presentation
Gestational age between 38 and 39 weeks verified by LMP and confirmed by first trimester ultrasound
Women diagnosed with GDM in the present pregnancy
No other contraindications to vaginal delivery

**Exclusion Criteria**

Pre-GDM
Prior C-section
Suspected estimated foetal weight > 4000 g at enrolment
Any known contraindications to vaginal delivery
Uncertain gestational age
Non reassuring foetal wellbeing necessitating delivery
Maternal pregnancy-related disease necessitating delivery
Bishop score > 7 at enrolment
Known foetal anomaly

If there are no other contraindications to deliver vaginally, patients will be enrolled between 38+0 and 39+0 weeks of gestational age (verified by last menstrual period (LMP) and confirmed by first trimester ultrasound). In the case of a discrepancy, gestational age assessed by ultrasound in the first trimester will be considered as the true reference. Uncertain gestational age or any major fetal anomaly will cause exclusion from the trial (Table 1). Women diagnosed with pre-gestational diabetes or demonstrating a prior C-section in their obstetrical history will be excluded. The identification of non-reassuring fetal status assessed by cardiotocography alone or as a formal part of a biophysical profile [22] or the presence of maternal or fetal conditions complicating pregnancy (e.g. severe preeclampsia/HELLP Syndrome or severe fetal growth restriction) and requiring immediate obstetrical intervention (prompt delivery or prompt C-section) will lead to the patient's exclusion.

After careful evaluation of inclusion and exclusion criteria (Table 1), suitable patients will have been identified. On the day of enrolment, an ultrasound assessment of fetal weight, as well as a Bishop score evaluation, will be performed. Whenever the estimated fetal weight exceeds 4000 gr, patients will be excluded from the trial and offered a C-section. A Bishop score greater than 7 will automatically exclude the patient from the trial. Furthermore, an attentive evaluation of possible contraindications to vaginal delivery and an assessment of fetal wellbeing through a cardiotocographic trace will be carried out at enrolment. The remaining inclusion/exclusion criteria will be assessed through the evaluation of the patient's current and past obstetric records.

Eligible women will be provided a comprehensive explanation of the project by the obstetrician on call, in terms of study procedures and risks versus benefits attached. Patients wishing to take part will be asked to sign a written consent form before being allocated to the randomization group.

**Figure 1:**
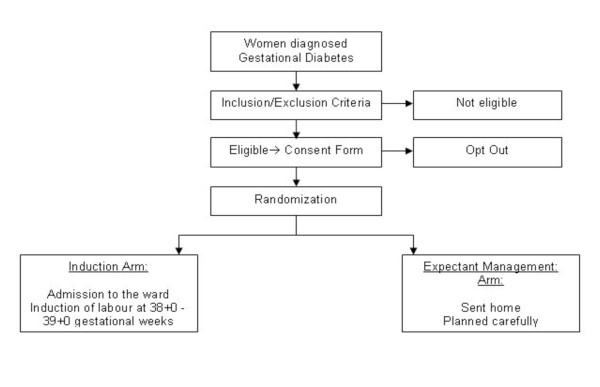
**Trial Flow chart**.

Data about obstetric history will be collected through consultation with the patient and evaluation of the patient's previous records and clinical reports. The outcomes will be evaluated during delivery and until maternal and neonatal discharge. Perinatal and maternal deaths will be identified until discharge. Perinatal deaths should include events up to 7 days after birth and most newborns will be discharged earlier. Our assumption is that perinatal death would occur almost exclusively in newborns manifesting complications during admission and consequently this could be accurately estimated by the time of discharge. At enrolment, patients assigned to the induction group will be admitted to the obstetric ward (Figure 1). Induction of labour will be performed by using dinoprostone, 2 mg vaginally, or dinoprostone, 0.5 mg intracervically, in 6-8 hour intervals (up to 5 doses) or dinoprostone, 10 mg vaginal device. Once the patient's Bishop score exceeds 7 or regular contractions are diagnosed, patients will be transferred to the delivery ward for artificial rupture of membranes (ARM) or oxytocin augmentation as indicated. Patients, in which cervical ripening does not occur (Bishop score < 7) after 5 attempts with Prostaglandin E2 (PGE2), will be offered either oxytocin or Foley catheter induction or C-section, according to local protocols.

Patients enrolled in the conservative management arm will be followed up twice weekly by Electronic Fetal Heart Rate monitoring and Biophysical profile (Figure 1). Patients will be followed up until 41+0 gestational weeks. Women, who will not deliver by this gestational age, will be admitted for labour induction (see the protocol above). Induction of labour will be offered when non-reassuring fetal status is suspected. All patients in the conservative arm will undergo fetal weight ultrasound estimation prior to induction. Patients with estimated fetal weight over 4000 gr will be offered a caesarean section.

General information on recruited patients, outcomes and randomization group will be indicated in an appropriate paper questionnaire in the first instance and then reported on an electronic database accessible from different study sites in the second instance. In each participating centre, medical personnel will fill up the electronic form directly. Data will be kept anonymous and will be analyzed by the Epidemiology and Biostatistics Unit - IRCCS Burlo Garofolo, using STATA 9 statistical package. Continuous variables will be reported as mean and standard deviation, categorical variables as proportions and percentages. The two groups will be compared by their main characteristics to evaluate the efficacy of the randomization process. To evaluate the main outcome of the study, C-section incidence will be compared between the two groups using the χ^2 ^test. Possible confounders such as: blood glucose control, obesity, participating centre policy etc. will be controlled through multivariate analysis (logistic regression). To compare secondary outcome variables between the two groups, the ANOVA technique will be used for continuous variables and χ^2 ^test for categorical. Statistically significant differences will be determined by a p-value inferior to 0.05. Although crossovers between the two allocation groups could complicate data interpretation, it is impossible to prevent them. However, data will be analysed by intention-to-treat. Finally, a sensitivity analysis will be carried out to confirm that results do not change significantly despite the variety of populations included in the study.

Data will be monitored by The Epidemiology and Biostatistics Unit at The Institute for Maternal and Child Health - IRCCS Burlo Garofolo. Serious adverse events will be reported to an apposite committee. This will consist of three senior obstetricians working at different recruiting centres who will independently verify that the adverse outcome has occurred, through assessing the patient's record. The Steering Committee for this trial will comprise Dr Salvatore Alberico (as chairman), Professor Moshe Hod and Professor Annunziata Lapolla.

Ethical approval of the protocol was obtained by Burlo Garofolo Ethical Committee (Coordinating Centre). Approval at each participating centre is required before starting the recruitment process and application for approval at each local Ethical Committee will take place in the next few months. The procedure will be explained in detail to recruited patients, who will be provided an information sheet and finally asked to sign a consent form if willing to participate.

## Discussion

The aim of the present study is to assess whether induction of labour at term would reduce C-section rate and ameliorate maternal and neonatal outcomes when compared to expectant management in pregnancies affected by GDM.

The main outcome of the study was planned to be a composite of main neonatal morbidity and mortality outcomes possibly related to GDM. The choice of creating a composite outcome derived from the practical impossibility of using shoulder dystocia alone as a single outcome. The rarity of this event would have precluded the possibility of carrying out the study, which would require a very large number of women to obtain significant results. The necessity of including very rare endpoints in a composite outcome in obstetrics research has been recently highlighted [23]. The attempt to include in a unique composite, the neonatal outcomes of interest, has unfortunately failed. In fact this process would have required combining very serious events, such neonatal death or shoulder dystocia, to neonatal hypoglycaemia or hyperbilirubinemia related to much less serious consequences, and for this reason unfeasible.

Therefore the main outcome of the study was decided to be the incidence of C-section among women undergoing induction of labour and those who will be assigned to expectant management with attentive maternal and fetal monitoring. This is a very relevant outcome both for women's health and for health service organizations. Furthermore, C-section rate was the main outcome of the only randomized controlled trial previously carried out on this topic [13].

Secondary outcomes of the study will be assessed at time of delivery and/or during maternal and neonatal admission until discharge. Outcomes evaluated at delivery are: gestational age, mode of delivery (spontaneous or operative vaginal delivery, C-section), spontaneous or instrumental third stage of labour, presence of perineal tears or performance of an episiotomy, postpartum haemorrhage (defined as bleeding from the genital tract of 1000 ml or more in the first 24 hours following the delivery of the baby) [20]. Furthermore, the occurrence of shoulder dystocia, defined as a delivery that requires additional obstetric manoeuvres to release the shoulders after gentle downward traction has failed, will be evaluated [24]. Shoulder dystocia occurs when either the anterior or, less commonly, the posterior fetal shoulder impacts on the maternal symphysis or sacral promontory [24] and its diagnosis will be confirmed by an apposite committee for serious adverse events. Serious adverse outcomes such as shoulder dystocia, fourth-degree perineal tears and uterine rupture will be reported to an independent data monitoring committee. Finally, the eventual manoeuvres performed to disengage the shoulder, newborn's birth weight, Apgar score at 1', 5' and 10' minutes, occurrence of arterial cord pH inferior to 7.02 will be recorded and evaluated.

The remaining secondary outcomes will be assessed until maternal and fetal discharge and are as follows: need for blood transfusion (whose main determinant should be the clinical picture and whose main therapeutic goals are to maintain haemoglobin > 8 g/dl, platelet count > 75 × 109/l, prothrombin < 1.5 × mean control, activated prothrombin times < 1.5 × mean control, fibrinogen > 1.0 g/l, when used in the most appropriate combination of intravenous clear fluids and blood products) [25], maternal or neonatal intensive care unit admission, duration and diagnosis, neonatal birth trauma (Erb's palsy, bone fracture, intra-cerebral/intra-ventricular haemorrhage, subdural hematoma), neonatal respiratory distress/transient tachypnea, neonatal need for respiratory support, neonatal hyperbilirubinemia, polycythemia or hypoglycaemia and maternal and perinatal death.

Analysis of neonatal plasma glucose level collected 1-2 h after delivery is performed at the local laboratory and results are provided to caregivers. Additional measurements of plasma glucose may be performed for clinical indications at the discretion of the attending physician, if signs or symptoms suggest sustained or later development of hypoglycemia. The need for other clinical assessments, such as bilirubin levels or neonatal respiratory function, is determined by specific clinical indications. All data regarding the assessment of potential neonatal morbidities are collected from the medical records of the newborn.

GDM is a common complication of pregnancy and could significantly affect fetal growth. Strong evidence supporting a specific management on timing of delivery of pregnant women with GDM is missing and there is no consensus on this matter. Expectant monitoring could determine an increase in macrosomic fetuses' incidence, leading to a higher C-section rate. Although induction of labour could prevent fetal macrosomia and its consequences, its performance is thought to be possibly related to an enhancement in C-section and instrumental vaginal delivery rates [26]. The aim of the present trial is to provide evidence on the best management of women at term, previously diagnosed with GDM, in terms of maternal and neonatal outcomes.

The GINEXMAL protocol and copies of all forms may be found on the following website: http://www.burlo.trieste.it. Additional file 1 - Patient information sheet and consent form, Additional file 2 - Patient recruitment form.

## List of Abbreviations

**ARM: **Artificial Rupture of Membranes; **C-section: **Caesarean Section; **GCT: **Glucose Challenge Test; **GINEXMAL: **Gestational Diabetes Induction Expectant Management of Labour; **GDM: **Gestational Diabetes Mellitus; **HAPO study: **The Hyperglycemia and Adverse Pregnancy Outcome Study; **LMP: **Last Menstrual Period; **OGCT: **Oral Glucose Challenge Test; **PGE2**: Prostaglandin E2;

## Competing interests

The authors declare there are not competing interests in the present research protocol.

## Authors' contributions

The authors mentioned separately with the corresponding author, equally contributed to conceptualize and design the research project and then write up the present protocol. The authors grouped under the name of "The GINEXMAL Research Group" contributed to correct and finalise the research protocol. We declare that all authors have read and approved this final manuscript.

## Pre-publication history

The pre-publication history for this paper can be accessed here:

http://www.biomedcentral.com/1471-2393/11/31/prepub

## Supplementary Material

Additional file 1 **Patient information sheet and Consent form**. Leaflet given to patients to explain what the project is about and form need to be signed before randomization if they agree to participate. Click here for file

Additional file 2 **Patient recruitment form**. Form to collect data about maternal and neonatal outcome for each randomized patient. Click here for file
