# Supplementary material for: "GINEXMAL RCT: Induction of labour versus expectant management in gestational diabetes pregnancies"
Source: BMC Pregnancy Childbirth. 2011 Apr 20;11:31. doi: 10.1186/1471-2393-11-31 (PMC3108319; doi:10.1186/1471-2393-11-31)
Supplement: Additional file 1 — Patient information sheet and Consent form. Leaflet given to patients to explain what the project is about and form need to be signed before randomization if they agree to participate. [file 1471-2393-11-31-S1.PDF]

BURLO

"GINEXMAL RCT: Induction of Labour versus Expectant Management in Gestational Diabetes Pregnancies"

Responsible: Dr. Salvatore Alberico, S.C. Patologia Ostetrica - Trieste
Prof. Moshe Hod, Rabin Medical Centre – Tel Aviv

INFORMATION SHEET

WHAT IS A CLINICAL STUDY?
It's a research project whose aim is to find a solution to unsolved medical problems. The objective is to explore the lack of knowledge in the use of drugs, features of diseases and importance to assume certain behaviours. The study is designed following the rules considered as valid by the international scientific community. In fact study methods are declared before the beginning of the study and this guarantees the validity and credibility of its results. The final aim is to achieve advantages for the population and so hypothesised benefits must be superior to risks. To guarantee that the study respects the scientific correctness, every project is evaluated and approved by a Scientific Committee. To maximise participants' awareness and safeness, every project undergoes the evaluation of an Ethical Committee (citizen representatives and experts in bioethics, medicine, pharmacology, law, biostatistics…).

WHAT IS THE STUDY AIM?
To understand whether in women diagnosed gestational diabetes at term (38-39 gestational weeks) would be better to induct labour or expect the spontaneous beginning of labour. The scientific literature doesn't provide useful information on this topic and so it is not clear if labour induction could take benefits to women's and their babies' health (reducing for example the risk of an excessive foetal weight gain). At the moment the choice between the two delivery modalities is left to single professionals working in maternities.

WHY WAS OFFERED TO ME TO PARTICIPATE?
It was offered to you because you were diagnosed gestational diabetes and you are at term of pregnancy without particular problems.

HOW MANY OTHER PATIENTS WILL PARTICIPATE AND FOR HOW LONG?
The study will be carried out in several centres in Italy and abroad. It has been planned to involve 1000 pregnant women with gestational diabetes at term. They will be followed-up from recruitment to hospital discharge of mother and baby.

WHO IS THE PRINCIPAL INVESTIGATOR PROMOTING THE STUDY?
The study is promoted, organized and coordinated by Dr Salvatore Alberico, Director of Obstetric Pathology Unit at Maternal and Child Health Institute IRCCS Burlo Garofalo (Trieste, Italy) and Prof Moshe Hod, Director of Maternal-Foetal Medicine Unit at Rabin Medical Centre (Tel Aviv, Israel)

ARE THERE ANY ECONOMICAL INTERESTS INVOLVED IN THE STUDY?
There are no economical interests involved in the study by either the study promoters or collaborators. The only aim of the study is the evaluation of the best approach to labour for pregnant women with gestational diabetes.

AM I OBLIGED TO TAKE PART?
The participation is voluntary so there are no obligations to take part.

WHAT WILL HAPPEN TO ME IF I DECIDE TO PARTICIPATE?
If you decide to take part you will be randomly assigned to one of the two groups: labour induction group or expectant management. If you are assigned to the induction group, you will be admitted to our Hospital and, under medical control, you will be administered every 6-8 hours a vaginal drug (prostaglandins) with the aim of inducting labour. In case labour starts you will be transferred to the labour ward for delivery exploitation. In case, after five administrations of the vaginal drug, labour doesn't start you will be offered an alternative method of induction using endovenous

oxytocin or Foley catheter or Caesarean delivery, based on call doctor's choice, in the best interest of you and your baby. If you are assigned to the expectant group, a foetal wellbeing assessment (ultrasound scan and cardiotocography) will be performed at our hospital twice a week up to 41 gestational weeks. In absence of any signs of spontaneous delivery by 41 weeks, labour will be inducted following the protocol described above. In presence of non-reassuring foetal wellbeing at one of the follow-ups, you will immediately be offered labour induction.

WHAT WILL HAPPEN IF I DECIDE NOT TO TAKE PART?
You will undergo what normally is offered to women in your clinical conditions. This normal management establishes a more frequent follow-up in comparison to pregnant women without gestational diabetes and that the health professional who is in charge of your care decides what is better for you and your baby.

WILL I BE AT A GREATER RISK IF I PARTICIPATE?
There are not more risks deriving from participating to this study. In fact, the scientific evidence has not demonstrated yet which of the two approaches is better to achieve more benefits for women in your clinical condition.

WILL I HAVE ANY BENEFITS IF I TAKE PART?
You won't achieve any direct benefits from your participation. This study though, could largely influence the future management of women like you, having been diagnosed with gestational diabetes. These women could largely benefit from these findings.

WHAT WILL HAPPEN IF SOMETHING GOES WRONG?
As already mentioned, there are no further risks other than those deriving from the normal management of women with the same clinical condition as you. All patients taking part to the research studies carried out at this Institute though, will benefit of an adequate covering insurance.

HOW THE INFORMATION COLLECTED WILL BE USED?
The collected data will be treated unanimously and will be analysed only by researchers involved in the project. The research will be the object of a final report analysis. Your identity won't be ever mentioned in any research reports.

IF I'M INTERESTED, WILL IT BE POSSIBLE TO RECEIVE INFORMATION ABOUT NEWS & RESULTS?
Yes, you could contact the Principal Investigator of this study, Dr Salvatore Alberico, Director of the Obstetric Pathology Unit at the Maternal and Child Health Institute – IRCCS Burlo Garofalo (tel. 040.3785.303, alberico@burlo.trieste.it).

WHO COULD I TALK TO FOR MORE INFORMATION AND CLARIFICATIONS?
You could talk to the doctor on call, who will inform you and will answer to all your questions.

COULD I CHANGE MY MIND DURING THE STUDY?
It is possible to change your mind and leave the study at any moments. In case you decide to leave the project you will be assisted in the best possible way and you will undergo the normal management for patients in your clinical conditions.

WHAT SHOULD I DO PRACTICALLY IF I DECIDED TO TAKE PART TO THIS STUDY?
If you decide to participate, after reading carefully the consent form and after receiving appropriate and clear information on aims, benefits, risks and possible inconveniences of this research project, you should sign the consent form.

THANK YOU VERY MUCH FOR YOUR ATTENTION.

BURLO

"GINEXMAL RCT: Induction of Labour versus Expectant Management in Gestational Diabetes Pregnancies"

Responsible: Dr Salvatore Alberico, S.C. Patologia Ostetrica - Trieste
Prof. Moshe Hod, Rabin Medical Center – Tel Aviv

CONSENT FORM

Surname……….........……………………........Name……………....………………… …./....../…….
Date of birth

Born in : ....……………………………....……Resident in:………………………..................……

Address……….. ……………………………………………………..……....Phone N°: ……/……………

I CONFIRM that:

1. I have understood the nature, aim, benefits risks and possible inconveniences deriving from this research project on the base of the information sheet I have read;

2. My participation to this study is absolutely voluntary;

3. I have understood that I could decide not to take part and I could leave the research project at any times, without causing any problems;

4. I have understood that I will be informed about the medical procedures I will undergone and tests' results and that at any times it will be possible to contact the Principal Investigator Dr Salvatore Alberico for any problems or further questions at the Obstetrics Pathology Unit – IRCCS Burlo Garofalo (alberico@burlo.trieste.it).

5. I have understood, after reading the information sheet that the collected data will be used to write a final report and my identity won't be mentioned in any research reports.

READ & APPROVED: ______________________________ ___/___/______
Signature Date
