# Supplementary material for: "GINEXMAL RCT: Induction of labour versus expectant management in gestational diabetes pregnancies"
Source: BMC Pregnancy Childbirth. 2011 Apr 20;11:31. doi: 10.1186/1471-2393-11-31 (PMC3108319; doi:10.1186/1471-2393-11-31)
Supplement: Additional file 2 — Patient recruitment form. Form to collect data about maternal and neonatal outcome for each randomized patient. [file 1471-2393-11-31-S2.PDF]

"GINEXMAL RCT: Induction of labour versus expectant management in gestational diabetes pregnancies."

Principal Investigators: Dr. Salvatore Alberico, S.C. Patologia Ostetrica – Trieste
Prof. Moshe Hod, Rabin Medical Centre– Tel Aviv

Verify the presence/absence of inclusion/exclusion criteria before performing randomization procedure!

Inclusion Criteria:

MATERNAL AGE ≥ 18 years	YES	NO
SINGLETON PREGNANCY	YES	NO
VERTEX PRESENTATION	YES	NO
GESTATIONAL AGE 38-39 weeks	YES	NO
DIAGNOSIS of GESTATIONAL DIABETES	YES	NO

→ **Exclude woman from the study if any NO is selected**

Exclusion Criteria:

PRE-GESTATIONAL DIABETES	YES	NO
PRIOR C-SECTION	YES	NO
ESTIMATED FETAL WEIGHT > 4000 gr	YES	NO
BISHOP SCORE > 7	YES	NO
UNCERTAIN GESTATIONAL AGE	YES	NO
CONTROINDICATIONS for VAGINAL DELIVERY	YES	NO
CHRONIC OR ACUTE FOETAL DISTRESS	YES	NO
MATERNAL DISEASE	YES	NO
MAJOR FOETAL MALFORMATION	YES	NO

→ **Exclude woman from the study if any YES is selected**

If women fulfilled all criteria for inclusion, perform **randomization procedure and fill in the form:**

GENERAL INFORMATION

☐ **TRIESTE** ☐ **TEL AVIV** ☐ **COLOMBO** ☐ **FLORENCE** ☐ **TURIN**
☐ **LUBJANA** ☐ **BRESCIA** ☐ **VENICE** ☐ **SAN DANIELE**

Envelope Number: ………… **Progressive number of recruitment:** ……… **Date of enrolment:** …/…/…
Patient's initials: ……………… **Date of birth:** …/…/… **Maternal Education** (number in yrs): ………….

Race: ☐ Caucasian ☐ Hispanic ☐ Asian ☐ Black

OBSTETRIC HISTORY

Gravida……… **Para**………
In one of the previous pregnancies: GDM: ☐ yes ☐ no; **Macrosomia**: ☐ yes ☐ no; **Dystocia:** ☐ yes ☐ no; **If YES, Type:** ☐ Lack of efficacy in labour induction ☐ Prolonged latent phase ☐ Primary dysfunctional labour ☐ Secondary arrest ☐ Unknown; **Shoulder Dystocia:** ☐ yes ☐ no; **Others:**…………………………………..

CURRENT PREGNANCY

LMP: … /…. /… **Confirmed:** □ yes □ no
If NOT: Ultrasound dating: …/…/… **EDD:** …/…/…
Height (m): ………………… **Pre-pregnancy weight (Kg):** ………… **Gestational Weight Gain (Kg):** ……………

GDM Diagnosis:
GCT date: …/…/… **Glucose load 50g:** 0'………… 60' ……………
OGTT date: …/…/… **Glucose load:** □ 75g □ 100g: 0'………. 60'………. 120'……. 180'……..
Fasting glucose: …… **Date:** …/…/….
HAPO study criteria □ yes □ no

GDM Control:
Well-Controlled □ yes □ no

GDM Treatment:
Insulin therapy: □ yes □ no; **If YES, Starting date:** …/…/…
N° of doses/day: □ rapid……….. □ intermediate……….; **Total units:** …………..; **Others:** ……………………………
Other Hypoglycaemic Drug: □ yes □ no; **If YES, what:** ……………………………….; **N° of doses/day:** ……………

Ultrasound with foetal growth acceleration: □ yes □ no; **date:** …/…/…
Estimated foetal weight (grams): ……………
LGA: □ yes □ no

DELIVERY

Induction: □ yes □ no

IF INDUCTION GROUP ->
Maternal Weight: (day of induction) ………… kg **Date of first induction:** …/…/… **Time:** …………….
Prostaglandins: □ yes □ no;
Attempt 1: □ Dinoprostone 10mg vaginal device □ Dinoprostone 0.5mg intracervically □ Dinoprostone 2mg vaginal □ Others (please specify):……………………………………………………………………….; **N° of doses:** ……………
Attempt 2: □ Dinoprostone 10mg vaginal device □ Dinoprostone 0.5mg intracervically □ Dinoprostone 2mg vaginal □ Others (please specify):……………………………………………………………………….; **N° of doses:** ……………
Attempt 3: □ Dinoprostone 10mg vaginal device □ Dinoprostone 0.5mg intracervically □ Dinoprostone 2mg vaginal □ Others (please specify):……………………………………………………………………….; **N° of doses:** ……………
Attempt 4: □ Dinoprostone 10mg vaginal device □ Dinoprostone 0.5mg intracervically □ Dinoprostone 2mg vaginal □ Others (please specify):……………………………………………………………………….; **N° of doses:** ……………
Attempt 5: □ Dinoprostone 10mg vaginal device □ Dinoprostone 0.5mg intracervically □ Dinoprostone 2mg vaginal □ Others (please specify):……………………………………………………………………….; **N° of doses:** ……………
Foley Catheter: □ yes □ no;
Oxytocin: □ yes □ no;
Membrane Sweeping □ yes □ no; **Artificial Rupture Membranes:** □ yes □ no; **PROM:** □ yes □ no;
If YES: Date: …/…/… **Time:** ………
Epidural: □ yes □ no;

IF EXPECTANT GROUP ->
Maternal Weight: (day of delivery)…………… **Total N° of foetal monitoring from enrolment:** ……….
Induction □ yes □ no; **If YES, Why:** □ Maternal Disease □ Foetal growth acceleration □ Others …………….
Date of first induction: …/…/… **Time:** …………

Prostaglandins: □ yes □ no;
Attempt 1: □ Dinoprostone 10mg vaginal device □ Dinoprostone 0.5mg intracervically □ Dinoprostone 2mg vaginal □ Others (please specify):……………………………………………………………………; **N° of doses:** ....
Attempt 2: □ Dinoprostone 10mg vaginal device □ Dinoprostone 0.5mg intracervically □ Dinoprostone 2mg vaginal □ Others (please specify):……………………………………………………………………; **N° of doses:** ....
Attempt 3: □ Dinoprostone 10mg vaginal device □ Dinoprostone 0.5mg intracervically □ Dinoprostone 2mg vaginal □ Others (please specify):……………………………………………………………………; **N° of doses:** ....
Attempt 4: □ Dinoprostone 10mg vaginal device □ Dinoprostone 0.5mg intracervically □ Dinoprostone 2mg vaginal □ Others (please specify):……………………………………………………………………; **N° of doses:** ....
Attempt 5: □ Dinoprostone 10mg vaginal device □ Dinoprostone 0.5mg intracervically □ Dinoprostone 2mg vaginal □ Others (please specify):……………………………………………………………………; **N° of doses:** ....
Oxytocin: □ yes □ no;
Foley Catheter: □ yes □ no;
Membrane Sweeping □ yes □ no; **Artificial Rupture Membranes**: □ yes □ no; **PROM:** □ yes □ no;
If YES: Date: …/…/… **Time**: ………
Epidural: □ yes □ no

Onset of Labour:
Date: …/…/… **Time:** ……………; **Antibiotics therapy in labour** □ yes □ no;

Delivery:
Date: …/…/… **Time:** ……………;
***GA* at delivery**: weeks… + days ……;
Mode: □ Spontaneous □ Vacuum □ Forceps □ CS
CS indication: □ acute foetal distress □ chronic foetal distress □ maternal disease □ mechanical dystocia □ dynamic dystocia □ placental abruption □ umbilical cord prolapse □ Others:……………………………………………; Foetal macrosomia □ yes □ no;

Maternal outcome:
Third stage: □ Spontaneous □ Instrumental
Perineum: □ Intact □ I grade perineal tears □ II grade perineal tears □ III grade perineal tears □ IV grade perineal tears □ Episiotomy □ Others:……………………………………………

PPH: □ yes □ no; **Blood transfusion:** □ yes □ no; **ICU Admission:** □ yes □ no; **If YES, Admission date:** …/…/… **Discharge date:** …/…/…; **Others:** ………………………………………………………………………………………………

Neonatal outcome:
Sex: □female □ male; **Weight** (Kg): ....; **Apgar score:** 1'……… 5'……… 10'………
Shoulder dystocia: □ yes □ no; **Manoeuvres:** □ None □ McRoberts □ McRoberts + Soprapubic Pressure □ Internal Manipulations (all-fours-position) □ Zavanelli
NICU Admission: □ yes □ no; **If YES, Admission date:** …/…/… **Discharge date:** …/…/…

Perinatal Death: □ yes □ no; **Arterial cord pH <7.02:** □ yes □ no; **Hyperbilirubinemia:** □ yes □ no; **Clinical neonatal hypoglycaemia:** □ yes □ no; **Biochemical neonatal hypoglycaemia:** □ yes □ no; **Polycythemia:** □ yes □ no; **Birth trauma:** □ None □ Herb palsy □ Bone fractures □ Intra-cerebral/intra-ventricular haemorrhage □ Subdural hematoma; **Respiratory distress/transient tachypnea:** □ yes □ no; **Need for respiratory support:** □ yes □ no; **Others:** ……………………………………………
